# Genome wide analysis of circulating miRNAs in growth hormone secreting pituitary neuroendocrine tumor patients’ plasma

**DOI:** 10.3389/fonc.2022.894317

**Published:** 2022-09-09

**Authors:** Helvijs Niedra, Raitis Peculis, Helena Daiga Litvina, Kaspars Megnis, Ilona Mandrika, Inga Balcere, Mihails Romanovs, Liva Steina, Janis Stukens, Austra Breiksa, Jurijs Nazarovs, Jelizaveta Sokolovska, Rasa Liutkeviciene, Alvita Vilkevicute, Ilze Konrade, Vita Rovite

**Affiliations:** ^1^ Department of molecular and functional genomics, Latvian Biomedical Research and Study Centre, Riga, Latvia; ^2^ Department of Endocrinology, Riga East Clinical University Hospital, Riga, Latvia; ^3^ Department of Internal Diseases, Riga Stradins University, Riga, Latvia; ^4^ Department of Neurosurgery, Faculty of Medicine Pauls Stradins Clinical University Hospital, Riga, Latvia; ^5^ Faculty of Medicine, University of Latvia Faculty of Medicine, Riga, Latvia; ^6^ Institute of Neuroscience, Lithuanian University of Health Sciences, Kaunas, Lithuania

**Keywords:** micro-RNA differential expression, circulating plasma micro-RNAs, growth hormone secreting pituitary neuroendocrine tumor, acromegaly, somatostatin analogue treatment

## Abstract

**Background:**

Circulating plasma miRNAs have been increasingly studied in the field of pituitary neuroendocrine tumor (PitNET) research. Our aim was to discover circulating plasma miRNAs species associated with growth hormone (GH) secreting PitNETs versus assess how the plasma levels of discovered miRNA candidates are impacted by SSA therapy and whether there is a difference in their levels between GH secreting PitNETs versus other PitNET types and healthy individuals.

**Design:**

We compared plasma miRNA content and levels before and after surgery focusing on GH secreting PitNET patients. Selected miRNA candidates from our data and literature were then tested in a longitudinal manner in somatostatin analogues (SSA) treatment group. Additionally, we validated selected targets in an independent GH secreting PitNET group.

**Methods:**

miRNA candidates were discovered using the whole miRNA sequencing approach and differential expression analysis. Selected miRNAs were then analyzed using real-time polymerase chain reaction (qPCR).

**Results:**

Whole miRNA sequencing discovered a total of 16 differentially expressed miRNAs (DEMs) in GH secreting PitNET patients’ plasma 24 hours after surgery and 19 DEMs between GH secreting PitNET patients’ plasma and non-functioning (NF) PitNET patients’ plasma. Seven miRNAs were selected for further testing of which miR-625-5p, miR-503-5p miR-181a-2-3p and miR-130b-3p showed a significant downregulation in plasma after 1 month of SSA treatment. mir-625-5p was found to be significantly downregulated in plasma of GH secreting PitNET patients vs. NF PitNET patients. miR-625-5p alongside miR-130b-3p were also found to be downregulated in GH PitNETs compared to healthy individuals.

**Conclusions:**

Our study suggests that expression of plasma miRNAs miR-625-5p, miR-503-5p miR-181a-2-3p and miR-130b-3p in GH secreting PitNETs is affected by SSA treatment. Additionally, miR-625-5p can distinguish GH secreting PitNETs from other PitNET types and healthy controls warranting further research on these miRNAs for treatment efficacy.

## 1 Introduction

Pituitary neuroendocrine tumors (PitNETs) are sellar region tumors composed of neuroendocrine cells with a clonal origin (1). PitNETs have a very high prevalence rate of 10 – 14% in population, most of these cases are asymptomatic and classified as incidentalomas (2). However, 1 out of 1000 PitNET cases are clinically relevant (3). Of the clinically relevant cases approximately 14% are growth hormone (GH) secreting PitNETs (4) which have a prevalence rate in the population ranging from 3 to 13.7 cases per 100’000 with an annual incidence of 0.2 to 1,1 cases per 100’000 (5). Chronic GH hypersecretion results in acromegaly hence in 95% acromegaly cases there is a presence of GH secreting PitNET (5). Acromegaly is a disease with increased morbidity and mortality due to excessive bone growth, cardiovascular manifestations, metabolic disorders, and respiratory complications (6). To reduce the health risks associated with acromegaly it is important to diagnose it early and perform a treatment *via* surgery or somatostatin analogue (SSA) therapy (7). The diagnostics of GH secreting PitNETs related acromegaly includes combined measurements of insulin growth factor 1 (IGF-1) and GH measurements *via* oral glucose tolerance test (OGTT) approach. This is followed by magnetic resonance imaging (MRI) scans of the sellar region to confirm that the source of excess GH is PitNET (8). Even now the diagnosis of GH secreting PitNETs is a complicated task, since IGF-1 levels are affected by age, sex and BMI and can be elevated even with normal GH levels (8). As for the GH levels after suppression with OGTT the resulting levels may vary between assays used (9). MRI scans can yield inconclusive results when in cases of microadenomas as the sensitivity can vary between 60 - 80%. This can lead to false assumptions that the cause of acromegaly is ectopic and not PitNET related (10). Therefore, it is vital to develop new sensitive and minimally invasive biomarkers to improve the healthcare of GH secreting PitNET patients.

A widely studied biomarker in regards to tumors is circulating miRNAs (11). miRNAs are small (22 nt long) non-coding RNA molecules that are transcribed from miRNA loci in DNA or generated during mRNA splicing (12). The primary biological purpose of miRNAs is the post transcriptional gene expression regulation *via* the interaction of the 3’ UTR region of the transcribed mRNA which represses the translation of the targeted mRNA (12). For this reason, their expression has been widely studied in various types of cancer tissues as dysregulation of specific miRNAs such as miR-15 and let-7 as can contribute to the tumor development (13). In GH secreting PitNETs there are several studies describing the expression of miRNAs within tissue of these tumors (14–17). As a result, a number of miRNAs have been dis-covered to be dysregulated in GH secreting PitNETs tissues. miR-125b, miR-886-5p were discovered to be upregulated while miR-503, miR-198, miR-125a-5p, miR-524-5p, miR-630 were downregulated in tissues of GH secreting PitNET patients’ group that were responsive to SSA and lanreotide treatments (14). miR-107 has been shown to be overexpressed in GH secreting PitNETs tissues compared to non-functioning PitNETs (NF PitNETs) (15) and miR-184 has been shown to be overexpressed in GH secreting PitNETs tissues compared to normal pituitary tissues from autopsy (16). GH secreting tumors harboring germline mutations *AIP* gene are significantly more invasive and it was found that two proliferation promoting miRNAs (miR-34a and miR-145) were highly upregulated in tumors with AIP mutations compared to tumors without AIP mutations (17).

Recently several studies regarding circulating miRNAs in plasma of cancer patients have shown that some miRNAs have a potential to diagnose and assess the treatment outcome of the disease (11). According to the Pubmed database search, the majority of miRNA studies regarding PitNETs have been carried out in postoperative tumor tissue samples with few studies on the circulating miRNAs. Studies that have already analyzed circulating miRNAs in plasma of PitNET patients and have shown promising results for miR-143-3p in NF follicle stimulating hormone/luteinizing hormone (FSH/LH) PitNETs (18). miR-16-5p, miR-145-5p, miR-7g-5p (19), miR-7-5p (20) in adrenocorticotropic hormone (ACTH) secreting PitNETs, and miR-29c-3p in GH secreting PitNETs (21).

In this study we carried out two primary NGS analyses in the plasma of PitNET patients. In the first analysis we compared preoperative and postoperative plasma taken from GH, Prolactin (PRL), ACTH secreting and NF PitNET patients. In the second analysis we compared the GH secreting PitNET patients’ preoperative plasma against NF PitNET patients’ preoperative plasma. In total we discovered seven potential miRNAs which could be associated with GH secreting PitNETs. Using qPCR, we further tested these seven miRNAs in an independent GH secreting PitNET patient cohort and longitudinally evaluated how their levels in plasma were affected by SSA therapy which according to our knowledge has not been previously reported.

## 2 Materials and methods

### 2.1 Study design

**Figure 1:**
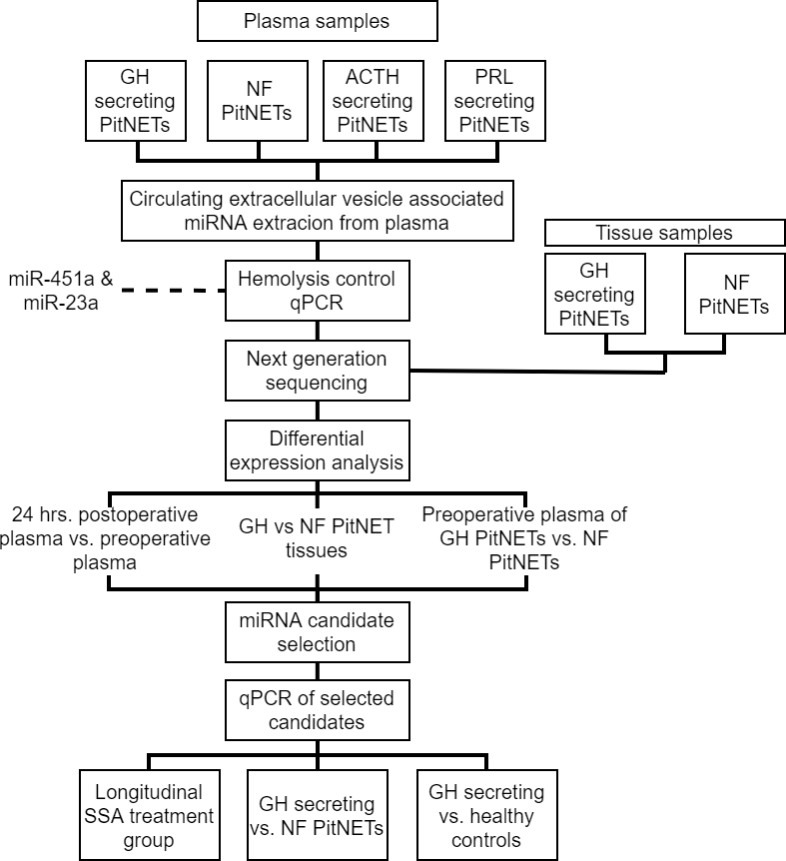
Flowchart explaining study design.

For NGS sequencing four ACTH, eight GH, six PRL secreting and 28 NF PitNETs patients were recruited and plasma samples were taken before and 24 hours after surgery (**Figure 1**). Following this samples underwent quality control by qPCR to evaluate the presence of miRNAs associated with the hemolysis. Samples that met the quality standards were sequenced by next generation sequencing. In differential expression analysis we compared the postoperative plasma against preoperative plasma for all four PitNET subtypes and we also compared the preoperative plasma from GH secreting PitNETs against NF PitNETs as our primary objective was to identify plasma miRNAs associated with GH secreting PitNETs. Additionally we sequenced three GH secreting PitNET and 10 NF PitNET tissue samples and carried out differential expression analysis by comparing the tissue of GH PitNETs against NF PitNETs. Differentially expressed miRNAs in plasma were compared with the literature findings and a panel of candidate miRNAs was designed for further validation by qPCR. We evaluated the candidate miRNA expression in plasma of six GH secreting PitNET patients receiving SSA treatment. In this group the plasma samples were taken before SSA treatment one, three and six months during the treatment. We also evaluated candidate miRNA expression in plasma of an independent GH secreting patient cohort (n = 15) from Lithuania against plasma from NF PitNETs (n = 5) and healthy controls (n = 13).

### 2.2 Subject recruitment

Patients were recruited from three institutions of Latvia and Lithuania: Riga East Clinical University hospital (RECUH, Latvia), Pauls Stradins Clinical University Hospital (PSCUH, (Latvia), and Lithuanian University of Health Sciences (LUHS, Lithuania). Inclusion criteria for all patients was: 18 of age and above and absence of other tumors. All patients had provided a written consent for participation in study and the study was approved by the Central Medical Ethics committee of Latvia (approval No.: 01-29.1/5035) and Lithuanian Ethics Committee for Biomedical Research (approval No.: P2-9/2003).

#### 2.2.1 NGS discovery cohort

For NGS study a total of 46 patients (eight GH secreting, 28 NF PitNETs, four ACTH secreting, six PRL secreting) over the age of 18 were recruited from Riga East Clinical University hospital and Pauls Stradins Clinical University Hospital (**Supplementary Table 1**). The NF PitNET group consisted of patients diagnosed with FSH/LH, immunonegative, silent GH, silent ACTH, and plurihormonal PitNETs, as our study mainly concentrated on miRNA species characteristic to GH secreting PitNETs and potential SSA effects, we did not subdivide NF PitNET in further subgroups Plasma samples were collected from all 46 patients in two time points: before surgery and 24 hours after surgery. 13 out of 46 included patients had available tumor tissue samples which were also included in this study (**Supplementary Table 1**). These 46 patients were recruited from RECUH and PSCUH. A detailed report on all NGS cohort patients’ clinical characteristics can be found in **Supplementary Table 1**.

#### 2.2.2 SSA treatment study cohort

For evaluation by qPCR in the SSA treatment group we recruited four GH secreting, one PRL secreting and one PRL/GH secreting (mammosomatotroph) PitNET patients who had received SSA therapy. Blood samples were collected in four time intervals: shortly before administration of SSA therapy, 1 month, 3 months and 6 months during SSA therapy. These six patients were also recruited from RECUH and PSCUH. A detailed report on all SSA treatment study cohort patients’ clinical characteristics can be found in **Supplementary Table 2**.

#### 2.2.3 GH secreting PitNETs vs. NF PitNETs study cohort and healthy controls cohort

Additionally, we also included 15 GH secreting PitNET patients from Institute of Neuroscience of LUHS for further NGS discovered plasma miRNA validation by qPCR in an independent GH secreting PitNET patient cohort. Firstly, the candidate miRNAs were evaluated GH secreting PitNETs vs. NF PitNETs setting. For NF PitNET cohort we included five NF PitNET patients which were recruited from both RECUH and PSCUH. Furthermore, we also evaluated the candidate miRNAs in GH vs. healthy controls setting. The plasma samples from healthy controls were acquired from Genome Database of Latvian Population (LGDB) (citation). A detailed report on GH study cohort patients’ clinical characteristics can be found in **Supplementary Table 3** and NF PitNET cohort in **Supplementary Table 4** and healthy control cohort in **Supplementary Table 5**.

### 2.3 Clinical sample processing

All blood samples were collected in 10 mL EDTA tubes. The tubes were inverted 10 times and kept at room temperature until further processing. Plasma layer was separated immediately upon blood sample collection using two-step centrifugation at room temperature: 1) 2000 RPM for 10 minutes, 2) 4000 RPM for 10 minutes. Plasma samples were aliquoted in 1 mL tubes and frozen at -80°C until further processing. This was done to ensure sufficient plasma quality for miRNA extraction. 13 (3 GH secreting and 10 NF PitNETs) out of 46 recruited patients had available fresh frozen tumor tissue samples for miRNA extraction.

Circulating extracellular vesicle bound miRNAs were extracted from 0,5 - 1 mL plasma samples using exoRNeasy Midi Kit (Qiagen, Germany) according to the manufacturer’s instructions. For circulating miRNA extraction, we used only aliquots from 2nd and 3rd EDTA vacutainers to avoid contamination of damaged cell nucleic acids during venipuncture. The extracted RNA was stored in two aliquots at -80°C - one for hemolysis control qPCR and one for NGS library preparation. Total RNA from fresh frozen PitNET tissue samples was extracted using AllPrep DNA/RNA/miRNA kit (Qiagen, Germany) according to manufacturer’s instructions. The extracted RNA was stored at -80°C.

### 2.4 Red blood cell hemolysis marker control of extracted circulating miRNA samples

All NGS discovery cohort patients’ plasma samples prior to library preparation for NGS underwent additional quality control for the presence of red blood cell hemolysis markers (miR-451a and miR-23a). This was done using qPCR on ViiA™ 7 (Appldied Biosystems) platform. The cDNA was synthesized using miRCURY LNA RT Kit (Qiagen, Germany) which was followed by SYBR Green based qPCR reaction setup using miRCURY LNA SYBR Green PCR Kit (Qiagen, Germany). Locked nucleic acid-based assays of miR-451a and miR-23a were designed and ordered through GeneGlobe (Qiagen, Germany). Following the qPCR, the ΔCt value of miR-23a - miR-451a was calculated. According to the manufacturer’s instructions ΔCt values higher than 5 indicate a risk of haemolysis and ΔCt values of 7 indicate that the samples are haemolysed. To improve the reliability of NGS data any samples with a ΔCt value greater than 5 were excluded from the library preparation part.

### 2.5 Library preparation for miRNA NGS analysis

Both plasma and tissue libraries for miRNA analysis by NGS were prepared using the Small RNA-Seq Library Prep Kit (Lexogen, Austria) according to the manufacturer’s instructions. To avoid contamination of adapter dimers and other RNAs out of small non-coding RNA size range we used BluePippin (Sage Science, USA) electrophoresis with a size range of 125 - 160 bp. The results were checked using a High Sensitivity DNA chip on Bioanalyzer 2100 (Agilent Technologies, USA). This ensured a total elimination of adapter dimers and the eluted fragments had peaks of around 144 and 153 bp. The final concentrations were checked using Qubit 2.0 dsDNA HS Kit (Thermo Fisher). The libraries were sequenced on Illumina MiSeq and NextSeq 500.

### 2.6 NGS data analysis

The FASTQ files from NGS were analyzed using CLC Genomics Workbench (v20.0.4). During read trimming any reads with Q (Phred score) < 30 were discarded. Reads also underwent trimming by length and any reads that did not fall into 15 - 55 nt length category were discarded. Trimmed reads were aligned to miRBase (V22) with the following settings: additional upstream/downstream bases = 2, missing upstream/downstream bases = 2, maximum mismatches = 2, sequence length = 15 to 28, minimum supporting count = 1. The counts were compiled and exported into DESeq2 compatible counts matrix format. miRNA differential expression analysis was done in DESeq2 (v1.30.1) in R (v 4.0.3) (22). The counts matrix used for this plasma analysis can be found in **Supplementary Table 6** and the following sample metadata can be found in **Supplementary Table 7**. Batch effect was estimated by constructing principal component analysis plots (**Supplementary Figure 1**). Upon generating DEseq2 model the following conditions were taken in account: sequencing batch, PitNET type (GH, ACTH, PRL, NF PitNET), surgery status (before surgery, 24 hours after surgery). For plasma samples a false discovery rate p value correction was done using fdrtool to achieve correct distribution of p values (**Supplementary Figure 2**). The counts matrix used to analyze tumor tissue samples can be found in **Supplementary Table 8** and the following metadata file in **Supplementary Table 9**. For tumor tissue analysis the following conditions were used: PitNET type (GH, NF PitNET), tumor size (macroadenoma, microadenoma). This time no sequencing batch was added since all samples were sequenced in one batch. Any miRNAs with less than five counts in less than three samples were excluded from differential expression analysis. Three following designs were used to evaluate the miRNA expression: 1) PitNET plasma 24 hrs. after surgery vs. before surgery (this was done for all PitNET subtypes separately), 2) GH secreting PitNET plasma before surgery vs NF PitNET plasma before surgery, 3) GH secreting PitNET tissues vs NF PitNET tissues. The R (4.0.3) script for DESeq2 analysis can be found in **Supplementary Figure 3**.

### 2.7 qPCR analysis of candidate miRNAs

For both SSA treatment study and GH secreting PitNETs vs. NF PitNETs study by qPCR the cDNA synthesis was carried out using miRCURY LNA RT kit (Qiagen, Germany). Following this a qPCR was carried out in triplicates using miRCURY LNA SYBR Green PCR Kit (Qiagen, Germany) and primers which were designed using GeneGlobe platform (Qiagen, Germany). Any wells within the replicates that had a variation in Ct values higher than 1 were excluded from calculating the average Ct. As a housekeeping gene we selected RNU6-1 (U6) small non-coding RNA. qPCR was carried out on the VIIA7 system (Thermo Fisher, USA). The fold changes were calculated using Livak’s method (ΔΔCt method) (23). Since miRNA expression in plasma can be affected by age, body mass index (BMI) and sex (24) the relationship between miRNA expression and clinical features was analyzed by constructing a multiple linear regression model in R (v 4.0.3). To evaluate miRNA expression in longitudinal SSA treatment group following clinical factors were assigned as independent variables: age, BMI, sex, SSA treatment (before treatment, 1 month during treatment, 3 months during treatment, 6 months during treatment). To evaluate miRNA expression in Lithuanian GH secreting PitNET against NF PitNETs and healthy controls the following clinical factors were assigned as independent variables: age, sex, BMI, treatment (SSA treatment, no treatment), tumor type (GH secreting, NF PitNET, no tumor).

## 3 Results

### 3.1 Quality control of NGS samples

**Figure 2:**
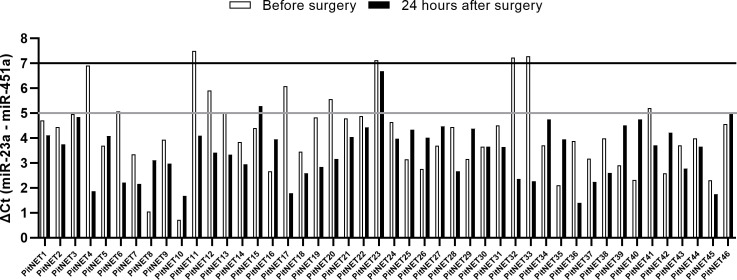
Results of miR-23a and miR-451a hemolysis test. ΔCt (miR-23a - miR-451a) < 5 indicates no presence of hemolysis, ΔCt between 5 and 7 indicates risk of hemolysis, ΔCt > 7 indicates that the sample is hemolyzed.

Total RNA from extracellular vesicles was extracted from 46 patients (**Supplementary Table 1**) before and after surgery plasma samples (92 samples in total). These samples underwent hemolysis test, which showed that four out of 46 patients had a ΔCt (miR-23a - miR-451a) value > 7 in either of the two samples indicating a presence of red blood cell hemolysis. Seven out of 46 patients had a ΔCt value between 5 and 7 in either of the two samples indicating a possible risk of hemolysis (**Figure 2**). These 11 patients were excluded from further analysis by NGS as there was a risk that they were contaminated with red blood cell associated miRNAs. The remaining 35 patients’ samples had ΔCt values < 5 indicating that the source of the extracted RNA is plasma and not hemolyzed erythrocytes (**Figure 2**).

### 3.2 Sequencing results of plasma and tumor tissue samples

**Figure 3:**
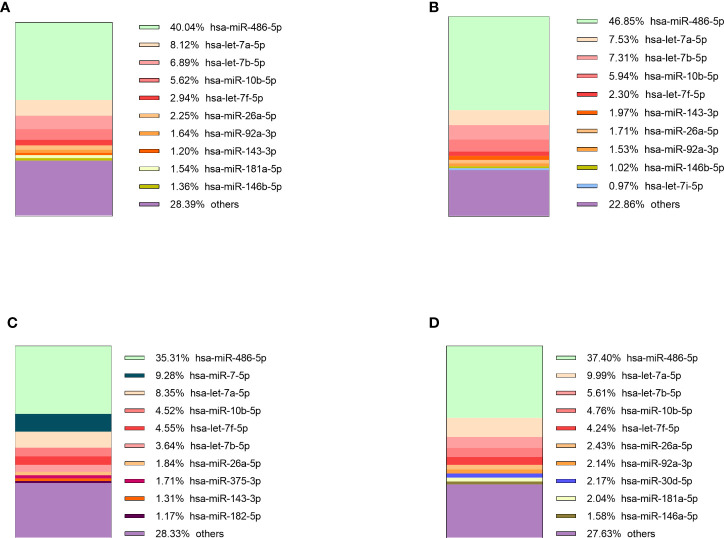
10 most expressed miRNAs in plasma of: **(A)** NF PitNET patients, **(B)** GH secreting PitNET patients, **(C)** PRL secreting PitNET patients, **(D)** ACTH secreting PitNET patients.

The sequencing of preoperative and postoperative plasma samples was performed for 35 patients (23 NF PitNETs, 6 GH secreting, 2 ACTH secreting and 4 PRL secreting), (70 samples in total) in six batches (**Supplementary Table 1**). On average 4.1 x 10^6^ (range: 1.5 x 10^6^ - 6.6 x 10^6^, stdev: 1.2 x 10^6^) reads per sample were acquired before trimming (**Supplementary Table 10**). After quality trimming the read count was reduced to a 2.6 x 10^6^ (range: 6.5 x 10^5^ - 4.2 x 10^6^, stdev: 8.2 x 10^5^) (**Supplementary Table 11**). Full report on sequence quality before and after trimming can be found on **Supplementary Figure 4**. Read annotation to miRBase yielded 2.1 x 10^5^ (7.8%) mature miRNA counts on average (range: 6.3 x 10^3^ - 1.9 x 10^6^, stdev: 2.4 x 10^5^). Compared to other samples the sample PitNET44 had unusually high annotated reads (1.9 x 10^6^, 50.6%) (**Supplementary Table 12**). Therefore, preoperative, and postoperative samples of this patient were excluded from further analysis. By analyzing the remaining 68 patients’ samples there was a significant difference in percentage of annotated miRNA counts between preoperative and postoperative plasma: 7.2% vs 6.2% (Student’s t-test P = 0.007). Five of the most common miRNAs across all PitNET types were: miR-486-5p, let-7a-5p, let-7b-5p, miR-10b-5p, and let-7f-5p (**Figure 3**).

**Figure 4:**
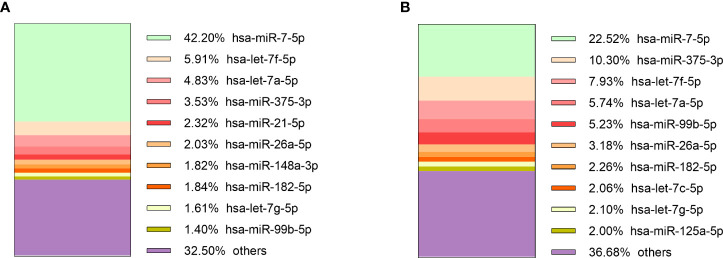
10 most expressed miRNAs in: **(A)** GH secreting PitNET tissues, **(B)** NF PitNET tissues.

Sequencing of tumor tissue samples was performed in 13 (10 NF and 3 GH) PitNETs (**Supplementary Table 1**). On average 7.4 x 10^6^ (range: 6.2 x 10^6^ - 8.7 x 10^6^, stdev: 7.3 x 10^5^) (**Supplementary Table 13**) raw reads were acquired before trimming. After trimming the read count was reduced to 6.7 x 10^6^ (range: 5.4 x 10^6^ - 7.4 x 10^6^, stdev: 5.6 x 10^5^) (**Supplementary Table 14**). Tumor tissue read annotation to miRBase yielded consistently higher annotated reads percentage than in plasma as on average 3.8 x 10^6^ (57.3%) reads were identified as mature miRNas (range: 1.2 x 10^6^ - 5.4 x 10^6^, stdev: 1.5 x 10^6^) (**Supplementary Table 15**). Five of the most expressed miRNAs in GH secreting PitNETs tissues were miR-7-5p, let-7f-5p, let-7a-5p, miR-375-3p, miR-21-5p (**Figure 4A**). In NF PitNETs tissues five of the most expressed miRNAs were miR-7-5p, miR-375-3p, let-7f-5p, let-7a-5p, miR-99b-5p (**Figure 4B**).

### 3.3 Differential expression analysis of postoperative vs. preoperative plasma

Following NGS differential expression analysis between preoperative and postoperative samples was carried out in 34 patients (after excluding 11 patients with insufficient quality of the plasma samples and one patient with abnormal read characteristics). The patients were categorized by tumor type: ACTH secreting (n = 2), GH secreting (n = 6), PRL secreting (n = 4), NF PitNETs (n = 22). In the NF PitNET group one differentially expressed miRNA (DEM) was detected (miR-200c-3p) which was downregulated 24 hours after surgery. In the GH secreting PitNET group a total of 16 DEMs were detected of which 10 were upregulated and 6 were downregulated in plasma 24 hours after surgery. In the GH secreting tumor group plasma, the most upregulated miRNA was miR-25-5p and the most downregulated miRNA was miR-503-5-p. In the PRL secreting PitNET group one DEM was detected (miR-205-5p) which was upregulated. In the ACTH secreting PitNET two DEMs were detected of which miR-141-3p was upregulated while miR-133a-3p was downregulated (**Table 1**).

**Table 1:** Results of differential expression analysis of postoperative vs. preoperative plasma from PitNET patients.

miRNA	PitNET	Log2FC (after vs before surgery)	P value	P adjusted (FDR)
miR-181b-5p	GH	0.89	4.4x 10^-5^	0.0052
miR-425-5p	GH	1.04	4.5 x 10^-5^	0.0052
miR-503-5p	GH	-6.00	4.6 x 10^-5^	0.0052
miR-181a-2-3p	GH	1.43	0.0001	0.0114
miR-142-3p	GH	-3.34	0.0002	0.0136
miR-1273h-3p	GH	3.20	0.0002	0.0136
miR-3615	GH	1.46	0.0002	0.0136
miR-148b-5p	GH	-4.28	0.0003	0.0136
miR-328-3p	GH	1.80	0.0004	0.0136
miR-345-5p	GH	1.72	0.0004	0.0136
miR-25-5p	GH	4.39	0.0009	0.0272
miR-95-3p	GH	-4.50	0.002	0.0421
miR-30c-5p	GH	-0.54	0.002	0.0421
miR-1843	GH	2.07	0.002	0.0421
miR-320b	GH	0.93	0.002	0.0423
miR-26b-3p	GH	-3.45	0.002	0.0423
miR-200c-3p	NFPA	-3.38	3.0 x 10^-6^	0.001
miR-141-3p	ACTH	23.54	2.2 x 10^-16^	7.5 x 10^-14^
miR-133a-3p	ACTH	-5.91	3.1 x 10^-05^	0.005
miR-205-5p	PRL	17.96	1.7x 10^-10^	5.8 x 10^-08^

PitNET, Pituitary neuroendocrine tumor; Log2FC, Log2 transformed fold change; FDR, False discovery rate; GH, Growth hormone secreting PitNET; NF PitNET, Non-functioning PitNET; ACTH, Adrenocorticotropic hormone secreting PitNET; PRL, Prolactin secreting PitNET.

### 3.4 Differential expression analysis of GH secreting PitNETs vs. NF PitNETs

**Figure 5:**
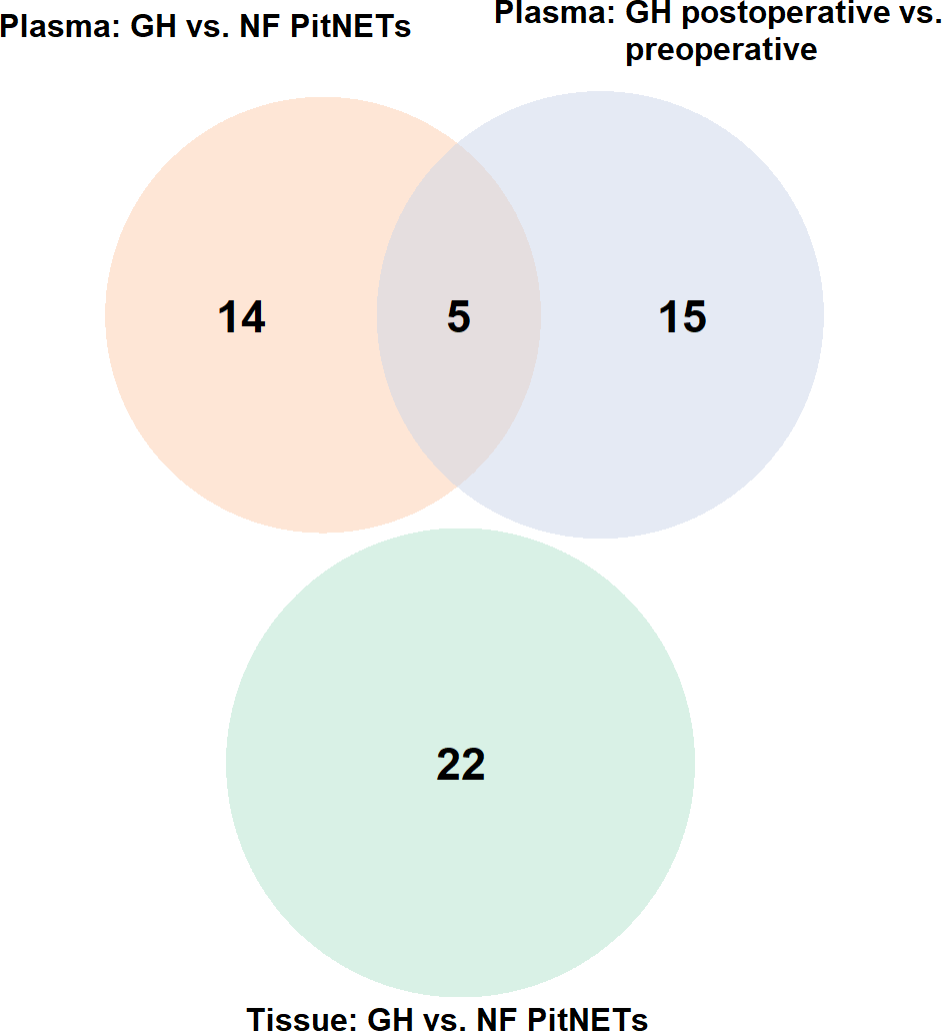
Venn diagram representing overlapping miRNAs between three differential expression analyses: (1) plasma of GH secreting PitNETs vs. NF PitNETs, (2) plasma of GH secreting PitNETs 24 hrs. after vs. before surgery, (3) tissue of GH PitNETs vs NF PitNETs.

Firstly compared the plasma before surgery taken from GH secreting PitNET patients against plasma before surgery taken from NF PitNET patients and as a result a total of 19 DEMs were identified. Of these four were upregulated while 15 were downregulated. The most upregulated miRNA was miR-503-5p while the most downregulated miRNA was miR-625-5p (**Table 2**). Of the 22 DEMs 5 DEMs (miR-181a-2-3p, miR-3615, miR-181b-5p, miR-503-5p, and miR-345-5p) were also differentially expressed in GH secreting PitNET patients’ plasma 24 hours after surgery. Secondly we compared the tissue samples of GH secreting PitNETs vs NF PitNETs. In this setting a total of 22 DEMs were identified of which 18 were downregulated and four were upregulated (**Table 3**). Interestingly, none of these DEMs overlapped with DEMs found in GH vs NF PitNET preoperative plasma or GH postoperative vs. preoperative plasma analyses (**Figure 5**).

**Table 2:** Results of differential expression analysis between preoperative GH secreting PitNET patients’ plasma vs. NF PitNET patients’ plasma.

miRNA	Log2FC	P value	P adjusted (FDR)
miR-4433b-5p	3.09	5.1 x 10^-6^	0.0017
miR-30a-5p	-0.95	1.6 x 10^-5^	0.0023
miR-222-3p	-1.51	2.1 x 10^-5^	0.0023
miR-181a-5p	-0.57	8.6 x 10^-5^	0.0050
miR-181a-2-3p	-1.34	8.7 x 10^-5^	0.0050
miR-3615	-1.44	9.0 x 10^-5^	0.0050
miR-181b-5p	-0.75	0.0001	0.0072
miR-503-5p	4.73	0.0002	0.0080
miR-335-5p	3.60	0.0002	0.0089
let-7d-3p	-1.59	0.0003	0.0107
miR-345-5p	-1.60	0.0003	0.0110
miR-323b-3p	-2.27	0.0005	0.0145
miR-16-2-3p	-1.89	0.0007	0.0188
miR-363-3p	-1.63	0.0009	0.0228
miR-485-3p	-2.07	0.0012	0.0281
miR-6852-5p	-1.86	0.0014	0.0292
let-7i-5p	0.59	0.0019	0.0373
miR-130b-3p	-2.10	0.0022	0.0419
miR-625-5p	-2.89	0.0024	0.0430

Log2FC, Log2 transformed fold change; FDR, False discovery rate.

**Table 3:** Results of differential expression analysis between GH secreting PitNET tissues vs. NF PitNET tissues.

miRNA	Log2FC	P value	P adjusted
miR-378a-3p	-3.01	3.64 x 10^-13^	2.21 x 10^-10^
miR-504-5p	-6.80	1.91 x 10^-8^	5.81 x 10^-6^
miR-501-3p	-3.97	5.20 x 10^-6^	0.0010
miR-21-5p	2.48	1.25 x 10^-5^	0.0019
miR-378d	-4.36	4.97 x 10^-5^	0.0045
miR-145-5p	-2.08	5.14 x 10-5	0.0045
miR-195-5p	2.82	5.22 x 10-5	0.0045
miR-378c	-5.20	7.09 x 10-5	0.0052
miR-195-3p	3.18	7.80 x 10-5	0.0052
miR-1271-5p	-2.68	9.57 x 10-5	0.0052
miR-1270	-6.02	0.0001	0.0052
miR-140-5p	-2.53	0.0001	0.0052
miR-33a-5p	-3.18	0.0002	0.0094
miR-149-3p	-5.58	0.0003	0.0112
miR-497-5p	2.83	0.0004	0.0175
miR-149-5p	-3.80	0.0005	0.0187
miR-204-5p	-3.61	0.0005	0.0187
miR-378g	-4.91	0.0009	0.0317
miR-137-5p	-5.38	0.0010	0.0317

Log2FC, Log2 transformed fold change.

### 3.5 Candidate miRNA selection for evaluation by qPCR

To select the candidate miRNAs for qPCR we combined the NGS results with literature findings regarding GH secreting PitNETs (**Table 4**). As a result, a total of seven miRNAs were selected for validation. Four miRNAs (miR-181b-5p, miR-181a-5p, miR-625-5p, miR-181a-2-3p) which were discovered to be dysregulated in our GH secreting PitNET datasets were reported in a study by Z. He (16). A microarray study reported that miR-503 and miR-30a were dysregulated in GH secreting PitNETs (14). Lastly qRT-PCR study reported that miR-130b was GH dysregulated GH secreting PitNETs (25). Accordingly, these seven miRNAs were selected as primary candidates for evaluation of their expression in plasma during SSA therapy of GH secreting PitNET patients’. Prior to this we also sequenced three available GH secreting PitNET and 10 NF PitNET tissue samples and checked for whether these miRNAs are expressed within the tumors (**Table 4**). Full report on miRNA counts in PitNET tissues can be accessed in **Supplementary Table 8**.

**Table 4:** Selection candidate miRNAs for further evaluation by qPCR.

miRNA	GH postoperative vs. preoperative plasma Log2FC	GH vs. NF PitNETs preoperative plasma Log2FC	Expressed in GH tissue	Reported in study
miR-181b-5p	0.89	-0.7	Yes	(16)
miR-181a-5p	–	-0.6	Yes	(16)
miR-625-5p	–	-2.9	Yes	(16)
miR-181a-2-3p	1.43	-1.3	Yes	(16)
miR-503-5p	-6.0	4.7	Yes	(14)
miR-130b-3p	–	-2.1	Yes	(25)
miR-30a-5p	–	-0.9	Yes	(14)

GH, Growth Hormone secreting; NF, non-functional; PitNETs, pituitary neuroendocrine tumors; Log2FC, Log2 transformed fold change.

### 3.6 qPCR testing of candidate miRNAs in plasma of GH secreting PitNET patients receiving SSA treatment

**Figure 6:**
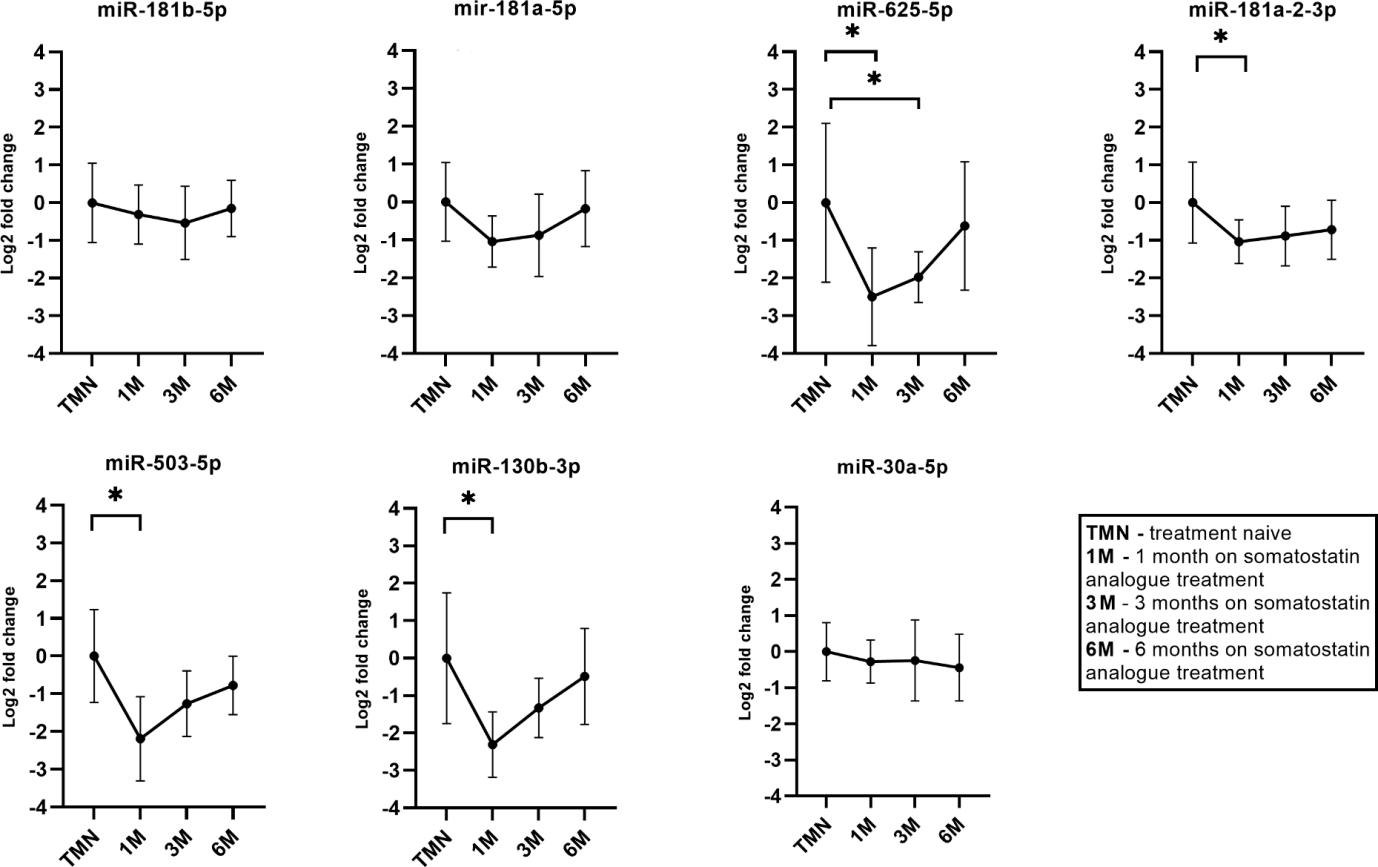
Expression by qPCR of seven candidate miRNAs before and during the somatostatin analogue (SSA) treatment. 1M – one month during SSA treatment, 3M – three months during SSA treatment, 6M - six months during SSA treatment. With “*” are marked changes with P < 0.05. P values were calculated using multiple linear regression by including the following factors: age, sex, body mass index, treatment duration.

In longitudinal SSA treatment group of six patients we observed that there was a decrease in plasma levels of all seven miRNAs (**Figure 6**). Within the first month during the therapy four miRNAs had statistically significant changes in expression (miR-625-5p, miR-181a-2-3p, miR-503-5p, miR-130b-3p). The miR-625-5p (Log2 FC = -2.48). Within the third month only miR-625-5p had a significant change in expression (Log2 FC = -1.97, P = 0.04). Within the sixth month the highest change was observed for miR-503-5p (Log2 FC = -0.78) however this change was not statistically significant. Interestingly the changes in expression for miR-181b-5p, miR-181a-5p and miR-30a-5p were associated with patients BMI and age. By analyzing all the data together, a dynamic can be observed where one month after the starting of SSA therapy has the highest decrease in these seven miRNAs and this change becomes less pronounced as therapy continues (**Figure 6**).

### 3.7 qPCR testing of candidate miRNAs in plasma of GH secreting PitNETs plasma vs. NF FSH/LH PitNETs plasma

**Figure 7:**
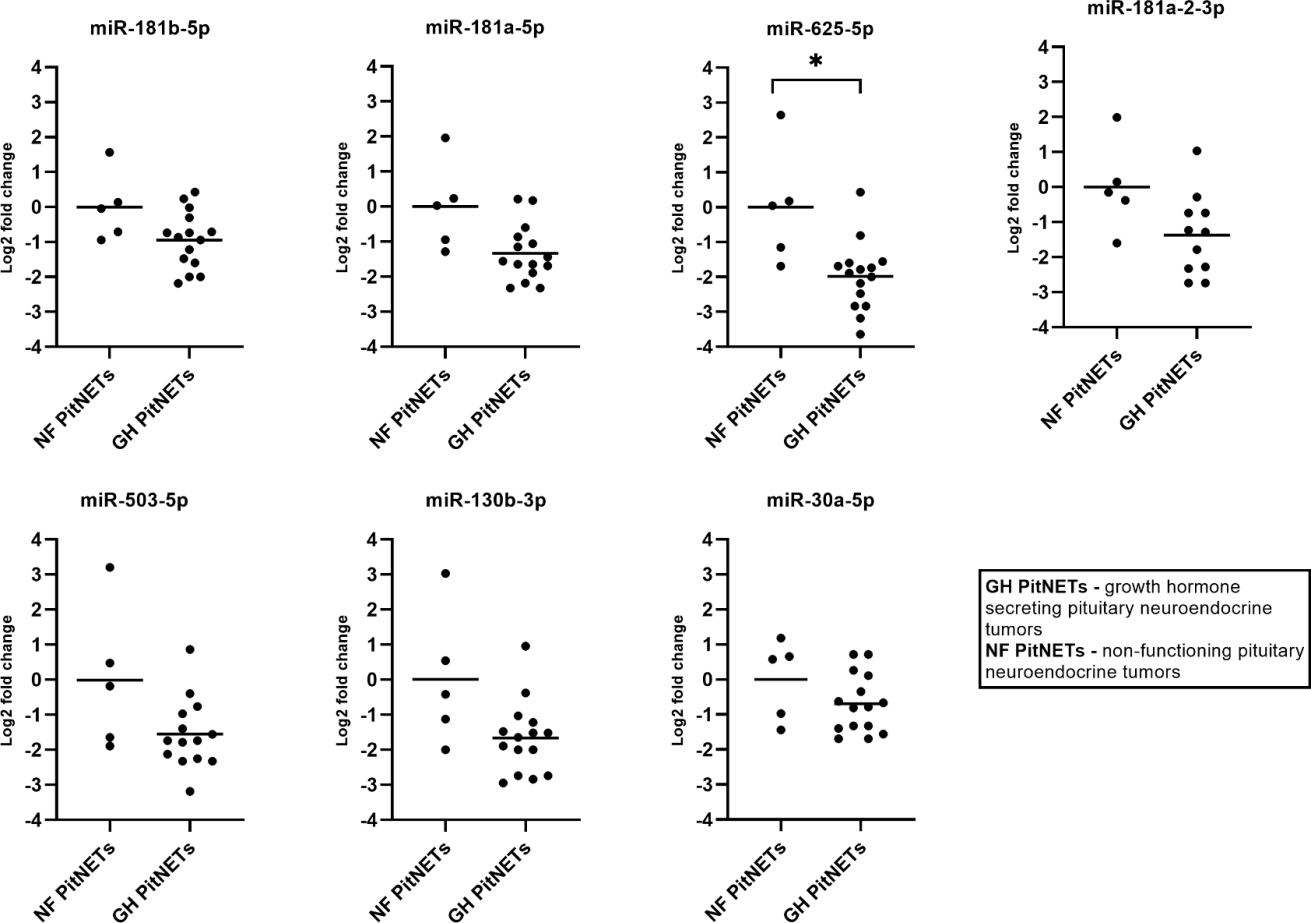
Expression by qPCR of seven candidate miRNAs in GH secreting PitNET patients’ plasma (n = 15) vs NF FSH/LH PitNET patients’ plasma (n = 5). With “*” are marked changes with P < 0.05. P values were calculated using multiple linear regression by including the following facots: age, sex, body mass index, tumor type, treatment (somatostatin analogue treatment, no treatment).

The seven miRNA candidates were evaluated in plasma of 15 GH secreting PitNET patients. Of these six patients had received SSA treatment while the remaining nine patients had no SSA treatment. The results on GH vs NF PitNETs are shown in **Figure 7**. By comparing patients with GH secreting PitNETs against patients NF-PitNETs only miR-625-5p had a significant change expression due to tumor type (Log2FC = -2.00, P = 0.0345). Interestingly all other clinical characteristics including SSA treatment had no impact on expression for all seven miRNAs. Regarding the direction of fold changes, the results are concordant with the NGS results, except for miR-503-5p which was downregulated in both qPCR testing groups but upregulated in the GH secreting vs NF PitNETs NGS discovery set, however the change was not statistically significant (**Table 2**).

### 3.8 qPCR testing of candidate miRNAs in plasma of GH secreting PitNET plasma vs. healthy controls plasma

**Figure 8:**
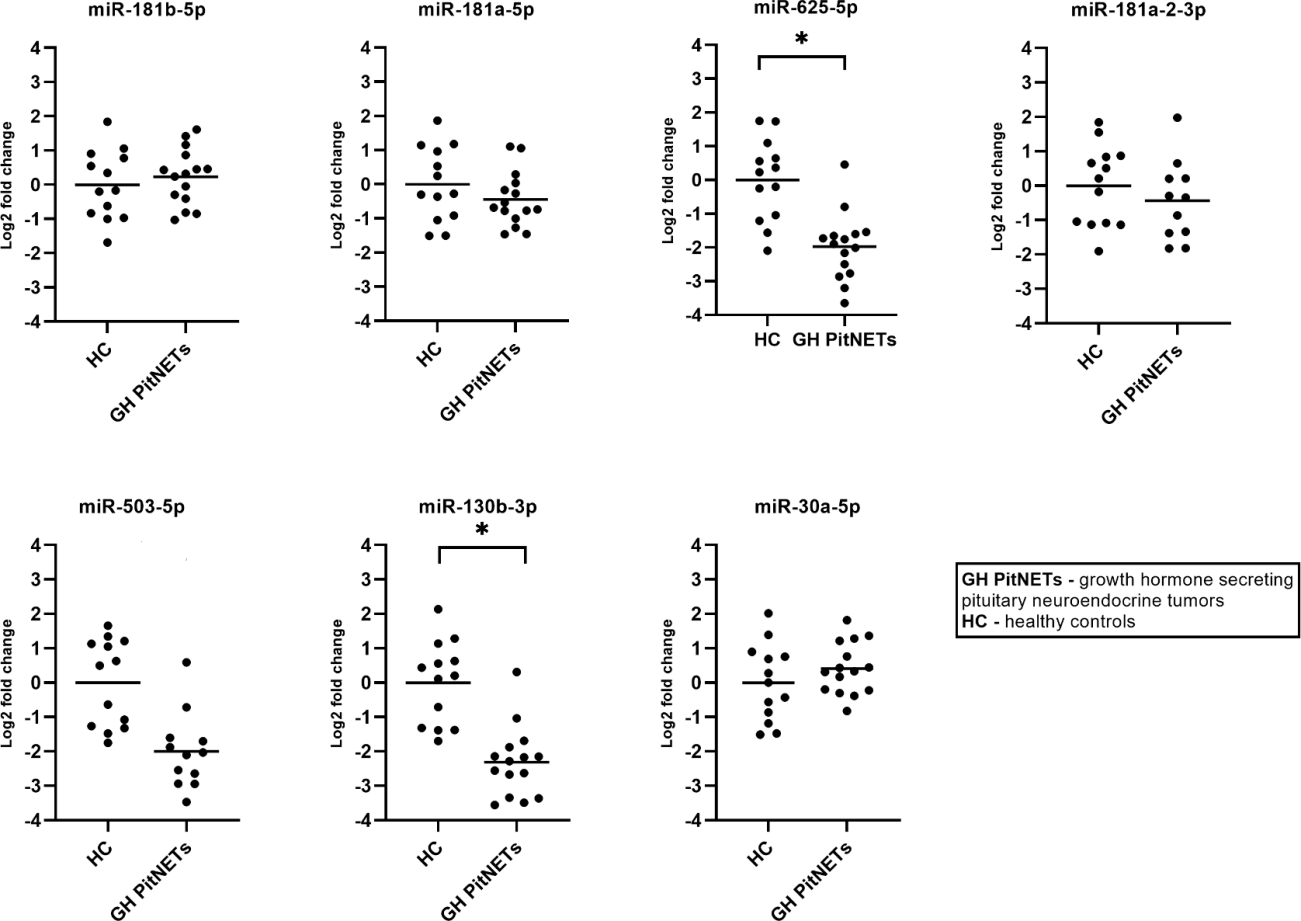
Expression by qPCR of seven candidate miRNAs in GH secreting PitNET patients’ plasma (n = 15) vs healthy subjects plasma (n = 13). With “*” are marked changes with P < 0.05. P values were calculated using multiple linear regression by including the following facots: age, sex, body mass index, presence of tumor, treatment (somatostatin analogue treatment, no treatment).

Lastly, we tested whether there are differences in plasma levels of cadidate miRNAs in 15 GH secreting PitNET patients vs. healthy controls. The healthy control group consisted of 13 subjects with average BMI, age and sex parameters matching with GH secreting PitNET cohort (**Supplementary Table 5**). In this scenario statistically significant changes in expression due to a presence of tumor were observed for two miRNAs (**Figure 8**) which were downregulated in GH secreting PitNETs: miR-625-5p (Log2FC = -1.98, P = 0.0009), miR-130b-3p (Log2FC = -2.31, P = 0.0002). While miR-503-5p and miR-30a-5p had also statistically significant changes (**Figure 8**) due to the presence of GH secreting tumor (Log2FC = -2.00, P = 0.001; Log2FC = 1.33, P = 0.046) these changes were also associated with patients’ BMI and age.

## 4 Discussion

In the field of PitNET research most miRNA studies have been carried out in tumor tissues or cell lines (14, 16, 26–30). The research of plasma miRNA studies in PitNETs is far scarcer (31). This can be attributed to the fact that the detection of tumor associated miRNAs in biofluids is methodologically more complex than in tumor tissue studies (32) and miRNA expressions bodily fluids can be also be affected by individuals age, sex and BMI (24). Despite this, three recent studies have focused the circulating miRNAs in plasma of PitNET patients showing that some miRNAs can serve as a potential markers of the disease: miR-16-5p, miR-145-5p, miR-7g-5p in ACTH secreting PitNETs (19), miR-143-3p in NF FSH/LH PitNETs (18), and miR-29c-3p in GH secreting PitNETs (21). There is also a dispute as to which is a better source to detect tumor associated miRNAs: whole plasma or extracellular vesicles isolated from plasma. Some studies have shown that isolating only extracellular vesicle fraction miRNAs may provide more consistent results in detecting tumor associated miRNAs (33, 34). The aim of this study was to evaluate the circulating extracellular vesicle associated miRNAs in plasma of PitNET patients before and after surgery as well as compare the plasma of GH secreting PitNET patients against other PitNET patients’ plasma to identify potential miRNAs species that are associated with GH secreting PitNETs. The identified miRNA candidates were then tested in an independent SSA therapy patient cohort and in GH secreting PitNET patient cohort.

One of the main technical risks in plasma miRNA sequencing studies including ours is blood sample haemolysis which can greatly alter the miRNA contents in plasma samples by spiking the plasma with miRNAs of erythrocyte origin (35). To avoid bias in miRNA discovery by NGS we performed quality control of all NGS plasma samples by qPCR profiling two haemolysis markers (miR-451a and miR-23a-3p) levels in plasma. As a result, plasma samples from 11 out of 46 patients were excluded from further analysis by NGS to improve the reliability of the results. Overall, these results indicate that blood sample collection and preparation is a vital part of any plasma miRNA related study to ensure sufficient sample size.

For preoperative vs. postoperative plasma analysis, we chose 24 hours after surgery for postoperative plasma collection. We based this design on the reasoning that miRNAs have a half-life up to 24 hours in bodily fluids (36) and at later collection times when the patient is discharged from hospital other uncontrolled factors such as altered diet (37) and infection (38) may impact the levels of miRNAs in plasma. Following the sequencing we performed two miRNA differential expression analyses for plasma samples 1) preoperative vs. postoperative plasma, 2) GH secreting PitNET preoperative plasma vs NF PitNET preoperative plasma. In post- vs. preoperative differential expression analysis we identified a total of 16 DEMs in GH secreting, two in ACTH secreting and one in PRL secreting PitNET groups. The DEMs identified in ACTH and PRL should be interpreted carefully as these two subgroups had small sample sizes (PRL n = 4, ACTH n = 2). In the NF PitNET subgroup only one DEM was identified (miR-200c-3p) which was downregulated after surgery. The downregulated miR-200c has been previously reported to be upregulated in PRL secreting PitNET tumor cell line (MMQ) and inhibits the apoptosis of tumor cells by targeting PTEN/Akt pathway confirming its tumor promoting role in PitNETs (39). Following study in MMQ cell line had shown that combination of bromocriptine and artesunate treatment reduces the expression miR-200c which increases the apoptosis of PitNET cells through upregulation of PTEN (40).

The overall aim of the following analyses was to identify miRNAs that are potentially associated with GH secreting tumors. For further validation by qPCR, we selected seven miRNAs from postoperative vs preoperative GH secreting PitNET plasma dataset and GH secreting vs. NF PitNETs plasma dataset. To reduce the number of candidates for validation we selected miRNAs that have been reported in previous studies regarding the PitNETs. In our study the miR-181b-5p was upregulated in GH patients’ plasma 24 hours after surgery and downregulated in GH patients’ plasma compared to NF PitNET patients’ plasma (**Table 4**). miR-181a-5p and miR-625-5p were downregulated in GH patients’ plasma compared to NF patients’ plasma. miR-181a-2-3p was upregulated in GH patients’ plasma 24 hours after surgery and downregulated in GH patients’ preoperative plasma compared to NF patients’ preoperative plasma (**Table 4**). These four miRNAs were previously reported in a NGS analysis by Z. He et al. where miR-181b-5p, miR-181a-5p, miR-625-5p and miR-181a-2-3p were reported to be downregulated in GH secreting tumors compared to normal pituitaries from autopsies (16). In a study by Z. G. Mao et al. miR-503 was reported to be downregulated in GH secreting tumors compared to normal pituitaries from autopsies (14). In our study the 5p variant of miR-503 was downregulated in GH patients’ plasma after surgery and upregulated in GH patients’ preoperative plasma compared to NF patients’. In our study we observed that the 3p variant of miR-130b is downregulated in GH patients’ preoperative plasma compared to NF patients’ (**Table 4**) while in a different study miR-130b was reported to be downregulated in GH and NF PitNETs compared to normal pituitaries from autopsies (25). Lastly, miR 30a-5p which was downregulated in GH secreting vs. NF PitNET plasma (**Table 4**) was also reported in a study by Z.G. Mao et al. where it was downregulated in GH secreting tumors compared to normal pituitaries (14). In another study it was found that the expression of miR-30a-5p was found to inversely correlate with the atypical morphological features and cavernous sinus invasion of the PitNETs (41). We also had available three tumor tissue samples from GH secreting PitNEts and 10 from NF PitNETs and following the sequencing we compared the miRNA expression of GH PitNETs against NF PitNETs. Initially we were hoping to identify some overlapping miRNAs with plasma as there have been studies reporting a positive correlation between levels of cell-free miRNAs and tumor tissue miRNAs (42, 43). However, none of the miRNAs that were dysregulated in GH secreting tumors plasma were overlapping with dysregulated miRNAs when we compared GH vs. NF PitNET tissues (**Figure 5**). One of the reasons might be the small sample size of only three GH secreting PitNET tissues.

Using qPCR we first tested longitudinally how SSA therapy affects expressions of seven chosen candidate miRNAs while also controlling for the following clinical characteristics: age, sex, BMI. It has been previously shown that usage of SSA treatment in neuroendocrine tumors can affect levels of certain miRNAs in bodily fluids (44). *In vitro* studies neuroendocrine tumor cell lines have shown that SSA treatment can effect expression levels of certain miRNAs in target cells (45). So far there has been only one study that has evaluated how SSA treatment affects levels of miRNAs in plasma of GH secreting PitNET patients showing promising results for miR-29c-3p (21). In our study we wanted to expand on this idea by evaluating the expression of the seven miRNA candidates (**Table 4**) longitudinally (before SSA treatment, 1, 3 and 6 months during the treatment). Additionally, the plasma taken before treatment and during the treatment came from the same individuals to avoid intraindividual variability. In the results we observed that all seven miRNAs were downregulated upon administration of SSA treatment (**Figure 6**). A statistically significant change was observed for miR-625-5p, miR-503-5p, miR-181a-2-3p and miR-130b-3p within first month of SSA treatment. Interestingly, this change became less pronounced with increased time of SSA administration. This could be due to the PitNET and treatment affected body tissue adaptation to the SSA treatment, but further studies are needed to confirm this hypothesis. Previous studies have shown that these four miRNAs are downregulated in GH secreting PitNETs compared to normal pituitaries (14, 16, 25) suggesting that they may have tumor suppressive role in GH secreting PitNETs. Despite this, studies in cancers have suggested that they also have tumor promoting roles. A study on malignant pleural mesothelioma has shown that patients with the tumor have elevated levels of miR-625-5p in plasma (46). While miR-625-5p showed a decrease after administration of SSA treatment (**Figure 6**) in our NGS results we did not observe a decrease of this miRNA in GH secreting PitNET patients’ plasma 24 hours after surgery. The reasoning behind such observation might be that SSA therapy may only affect the levels of this miRNA in plasma. This could also be due to the fact that 24 hours after surgery may not be enough to observe less pronounced changes in miRNA levels as the half-life of miRNAs can be up to 24 hours (36). In colorectal cancers it has been shown that upregulation miR-503-5p is associated with the deletion of p53 and increases resistance to oxaliplatin treatment (47). In our NGS results we observed a major decrease of miR-503-5p (Log2FC = -6.0) in plasma 24 hours after surgery and the decrease of miR-503-5p after SSA treatment could suggest that this miRNA could be directly associated with GH secreting PitNETs. However these results need to be interpreted with caution as there has been a study which showed that plasma miR-503-5p can be impacted by patient’s BMI (48) this was also shown when we compared GH secreting PitNETs vs healthy controls by qPCR. The reason why BMI did not show a significant impact on this miRNA in longitudinal SSA could be due to small sample size of six patients. Regarding miR-181a-2-3p it has been shown in gastric carcinoma cell lines that this miRNA is highly upregulated and promotes tumor growth by targeting MYLK (49). Interestingly the NGS results of before vs. after surgery are somewhat conflicting with the results of SSA therapy as this miRNA was upregulated (Log2FC = 1.43) in plasma 24 hours after surgery while it was downregulated in plasma during the administration of SSA treatment (**Figure 6**). Perhaps the upregulation of miR-181a-2-3p after surgery is due to the invasive nature of the procedure. miR-130b-3p is yet another miRNA that can promote tumor growth *in-vivo* by targeting *PTEN* and can be carried by exosomes in plasma (50). Increased levels of miR-130b-3p in plasma were observed in mice injected with miR-130b-3p overexpressing cells (50). However, in our NGS results we did not observe a decrease in plasma miR-130b-3p 24 hours after surgery. Nevertheless, the qPCR results in the SSA treatment group (**Figure 6**) suggest that miR-625-5p, miR-181a-2-3p, miR-130b-3p, and miR-503-5p are potential miRNAs to monitor SSA therapy and therefore require further functional evaluation.

Lastly, we tested whether these seven miRNAs can be used to distinguish between GH secreting PitNETs NF PitNETs and healthy controls. In case of GH secreting PitNETs it is vital to diagnose them early as prolonged exposure of GH secreting PitNET can cause comorbidities characteristic to acromegaly (51). It has been also shown that hormone secreting PitNETs are less likely to cause visual field impairment than NF PitNETs (52). Therefore, we believe there is a necessity for development of novel minimally invasive biomarkers that can distinguish GH secreting PitNETs from other PitNET subtypes. In this experiment we included 15 GH secreting PitNET patients from Lithuania (**Supplementary Table 3**). As a NF PitNET control group, we included five FSH/LH PitNET patients (**Supplementary Table 4**). As a healthy control group, we included 13 individuals with average BMI, age parameters and sex distribution matching with 15 Lithuanian GH secreting PitNET cohort. Interestingly, while 6 patients had received SSA treatment in our multiple linear regression model we did not observe any significant changes in miRNA expressions related to SSA treatment in both GH PitNETs vs NF PitNETs and GH PitNETs vs healthy controls. This could be due to the fact that the six patients from Lithuanian cohort had the SSA treatment for longer than one month and our results in longitudinal SSA treatment group showed that changes in plasma miRNAs were pronounced only within first month. Within GH PitNETs vs NF PitNETs we observed that all seven miRNAs are downregulated in both GH secreting PitNETs, and the fold changes of qPCR results are concordant with the NGS results (**Figure 7** and **Table 4**). Out of seven miRNAs only one miRNA (miR-625-5p) had statistically significant change in expression (**Figure 7**) due tumor type. Within GH PitNETs vs healthy controls, we observed changes in two miRNAs (miR-625-5p and miR-130b-3p) due to tumor type (**Figure 8**). Interestingly, compared to GH vs NF PitNETs and SSA treatment this time we could observe changes in expression (miR-503-5p and miR-30a-5p) due to BMI and age of the patients. This is further supported by a studies which showed that miR-30 family miRNAs are impacted by age (24) and miR-503 is impacted by BMI (48). Altogether these results show that plasma miR-625-5p could potentially be used to distinguish GH secreting PitNETs from both NF PitNETs and healthy controls and miR-130b-3p could potentially distinguish GH PitNETs from healthy controls.

A limitation of this study was the composition of the NF PitNET patient group for NGS analysis as at the time of diagnosis the clinicians and pathologists did not adhere to the 2017 WHO PitNET classification guidelines (53) for some of the patients. Full immunohistochemistry report compatible with 2017 WHO guidelines is available only for 11 out of 23 sequenced NF PitNET patients. As a result our NF PitNET subgroup was composed of FSH/LH tumors, immunonegative tumors and silent corticotroph (ACTH) and somatotroph (GH) tumors and plurihormonal tumors (54). However, as our primary goal was to search for markers that distinguish GH secreting PitNET from other PitNET types therefore this limitation did not affect our primary results significantly. We were also able to demonstrate that specific GH PitNET miRNAs are impacted by the SSA therapy. With further investigation they might be used for treatment efficacy evaluation in a minimally invasive manner.

In conclusion, this study shows that levels are affected by SSA treatment. This is also the first study that has compared the expression of plasma miRNAs between different PitNET subtypes. The results show that plasma levels miR-625-5p could potentially distinguish GH secreting PitNETs from other PitNET types and miR-625-5p and miR-130b-3p can distinguish patients with GH PitNETs from healthy subjects. We also demonstrate that SSA therapy could affect plasma levels of miRNAs characteristic to GH PitNETs (miR-625-5p, miR-181a-2-3p, miR-130b-3p, and miR-503-5p) warranting further studies of these miRNAS as blood-based markers in monitoring of treatment efficacy.

## Data availability statement

The next generation sequencing data used in this study can be found in the online repository the Gene Expression Omnibus at the following link - https://www.ncbi.nlm.nih.gov/geo/query/acc.cgi?acc=GSE196647. The source data used to generate figures and tables in this manuscript can be found in **Supplementary Material**.

## Ethics statement

The use of patients’ samples in this study was approved by the Central Medical Ethics committee of Latvia (approval No.: 01-29.1/5035) and Lithuanian Ethics Committee for Biomedical Research (approval No.: P2-9/2003). The patients/participants provided their written informed consent to participate in this study.

## Author contributions

HN: manuscript preparation, sample preparation, and bioinformatics analysis. RP: manuscript preparation. HDL and IM: sample preparation. KM: clinical samples management. IB, MR, LS, JSt, AB, JN, JSo, RL, AV, IK: patient recruitment and clinical data management. VR: manuscript preparation and correspondence. All authors contributed to the article and approved the submitted version.

## Funding

This research was funded by the European Regional Development Fund within the project RNA molecular determinants in development of pituitary adenoma” (1.1.1.1/18/A/089).

## Acknowledgments

This research was supported by the European Regional Development Fund within the project RNA molecular determinants in development of pituitary adenoma” (1.1.1.1/18/A/089). The authors acknowledge the Latvian Biomedical Research and Study Centre and the Genome Database of the Latvian Population for providing infrastructure, biological material and data.

## Conflict of interest

The authors declare that the research was conducted in the absence of any commercial or financial relationships that could be construed as a potential conflict of interest.

## Publisher’s note

All claims expressed in this article are solely those of the authors and do not necessarily represent those of their affiliated organizations, or those of the publisher, the editors and the reviewers. Any product that may be evaluated in this article, or claim that may be made by its manufacturer, is not guaranteed or endorsed by the publisher.

## References

[1] Herman V Fagin J Gonsky R Kovacs K Melmed S . Clonal origin of pituitary adenomas. J Clin Endocrinol Metab (1990) 71:1427–33. doi: 10.1210/jcem-71-6-1427 1977759

[2] Daly AF Beckers A . The epidemiology of pituitary adenomas. Endocrinol Metab Clin North Am (2020) 49:347–55. doi: 10.1016/j.ecl.2020.04.002 32741475

[3] van den Broek MFM van Nesselrooij BPM Verrijn Stuart AA van Leeuwaarde RS Valk GD . Clinical relevance of genetic analysis in patients with pituitary adenomas: A systematic review. Front Endocrinol (Lausanne) (2019) 10:837. doi: 10.3389/fendo.2019.00837 31920960 PMC6914701

[4] Day PF Loto MG Glerean M Picasso MFR Lovazzano S Giunta DH . Incidence and prevalence of clinically relevant pituitary adenomas: Retrospective cohort study in a health management organization in Buenos Aires, Argentina. Arch Endocrinol Metab (2016) 60:554–61. doi: 10.1590/2359-3997000000195 PMC10522164 27982201

[5] Chin SO . Epidemiology of functioning pituitary adenomas. Endocrinol Metab (Seoul Korea) (2020) 35:237–42. doi: 10.3803/EnM.2020.35.2.237 PMC7386114 32615708

[6] Zahr R Fleseriu M . Updates in diagnosis and treatment of acromegaly. Eur Endocrinol (2018) 14:57–61. doi: 10.17925/EE.2018.14.2.57 30349595 PMC6182922

[7] Adelman DT Liebert KJ Nachtigall LB Lamerson M Bakker B . Acromegaly: The disease, its impact on patients, and managing the burden of long-term treatment. Int J Gen Med (2013) 6:31–8. doi: 10.2147/IJGM.S38594 PMC3555549 23359786

[8] AlDallal S . Acromegaly: A challenging condition to diagnose. Int J Gen Med (2018) 11:337–43. doi: 10.2147/IJGM.S169611 PMC6112775 30197531

[9] Arafat AM Möhlig M Weickert MO Perschel FH Purschwitz J Spranger J . Growth hormone response during oral glucose tolerance test: The impact of assay method on the estimation of reference values in patients with acromegaly and in healthy controls, and the role of gender, age, and body mass index. J Clin Endocrinol Metab (2008) 93:1254–62. doi: 10.1210/jc.2007-2084 18171702

[10] Grober Y Grober H Wintermark M Jane JA Oldfield EH . Comparison of MRI techniques for detecting microadenomas in cushing’s disease. J Neurosurg (2018) 128:1051–7. doi: 10.3171/2017.3.JNS163122 28452619

[11] Cui M Wang H Yao X Zhang D Xie Y Cui R . Circulating MicroRNAs in cancer: Potential and challenge. Front Genet (2019) 10:626. doi: 10.3389/fgene.2019.00626 31379918 PMC6656856

[12] O’Brien J Hayder H Zayed Y Peng C . Overview of MicroRNA biogenesis, mechanisms of actions, and circulation. Front Endocrinol (Lausanne) (2018) 9:402. doi: 10.3389/fendo.2018.00402 30123182 PMC6085463

[13] Peng Y Croce CM . The role of microRNAs in human cancer. Signal Transduct Target Ther (2016) 1:15004. doi: 10.1038/sigtrans.2015.4 PMC5661652 29263891

[14] Mao Z-G He D-S Zhou J Yao B Xiao W-W Chen C-H . Differential expression of microRNAs in GH-secreting pituitary adenomas. Diagn Pathol (2010) 5:79. doi: 10.1186/1746-1596-5-79 21138567 PMC3017030

[15] Trivellin G Butz H Delhove J Igreja S Chahal HS Zivkovic V . MicroRNA miR-107 is overexpressed in pituitary adenomas and inhibits the expression of aryl hydrocarbon receptor-interacting protein *in vitro* . Am J Physiol - Endocrinol Metab (2012) 303:708–19. doi: 10.1152/ajpendo.00546.2011 22811466

[16] He Z Chen L Hu X Tang J He L Hu J . Next-generation sequencing of microRNAs reveals a unique expression pattern in different types of pituitary adenomas. Endocr J (2019) 66:709–22. doi: 10.1507/endocrj.EJ18-0487 31061247

[17] Bogner E-M Daly AF Gulde S Karhu A Irmler M Beckers J . miR-34a is upregulated in AIP-mutated somatotropinomas and promotes octreotide resistance. Int J Cancer (2020) 147:3523–38. doi: 10.1002/ijc.33268 32856736

[18] Németh K Darvasi O Likó I Szücs N Czirják S Reiniger L . Comprehensive analysis of circulating microRNAs in plasma of patients with pituitary adenomas. J Clin Endocrinol Metab (2019) 104 (9):4151–68. doi: 10.1210/jc.2018-02479 31112271

[19] Belaya Z Khandaeva P Nonn L Nikitin A Solodovnikov A Sitkin I . Circulating plasma microRNA to differentiate cushing’s disease from ectopic ACTH syndrome. Front Endocrinol (Lausanne) (2020) 11:331. doi: 10.3389/fendo.2020.00331 32582027 PMC7291947

[20] Niedra H Peculis R Konrade I Balcere I Romanovs M Steina L . Case report: Micro-RNAs in plasma from bilateral inferior petrosal sinus sampling and peripheral blood from corticotroph pituitary neuroendocrine tumors. Front Endocrinol (Lausanne) (2022) 13:748152. doi: 10.3389/fendo.2022.748152 35528014 PMC9072666

[21] Korkmaz H Hekimler Öztürk K Torus B . Circulating miR-29c-3p is downregulated in patients with acromegaly. Turkish J Med Sci (2021) 51:2081–6. doi: 10.3906/sag-2010-245 PMC8569748 34013701

[22] Love MI Huber W Anders S . Moderated estimation of fold change and dispersion for RNA-seq data with DESeq2. Genome Biol (2014) 15:550. doi: 10.1186/s13059-014-0550-8 25516281 PMC4302049

[23] Livak KJ Schmittgen TD . Analysis of relative gene expression data using real-time quantitative PCR and the 2(-delta delta C(T)) method. Methods (2001) 25:402–8. doi: 10.1006/meth.2001.1262 11846609

[24] Ameling S Kacprowski T Chilukoti RK Malsch C Liebscher V Suhre K . Associations of circulating plasma microRNAs with age, body mass index and sex in a population-based study. BMC Med Genomics (2015) 8:61. doi: 10.1186/s12920-015-0136-7 26462558 PMC4604724

[25] Leone V Langella C D’Angelo D Mussnich P Wierinckx A Terracciano L . Mir-23b and miR-130b expression is downregulated in pituitary adenomas. Mol Cell Endocrinol (2014) 390:1–7. doi: 10.1016/j.mce.2014.03.002 24681352

[26] Tang H Zhu D Zhang G Luo X Xie W . AFAP1-AS1 promotes proliferation of pituitary adenoma cells through miR-103a-3p to activate PI3K/AKT signaling pathway. World Neurosurg (2019) 130:e888–98. doi: 10.1016/j.wneu.2019.07.032 31299308

[27] Huang T Cai M Chen C Ling C Zhang B Zheng W . LINC01116 boosts the progression of pituitary adenoma *via* regulating miR-744-5p/HOXB8 pathway. Mol Cell Endocrinol (2021) 536:111350. doi: 10.1016/j.mce.2021.111350 34098015

[28] Boresowicz J Kober P Rusetska N Maksymowicz M Paziewska A Dąbrowska M . The search of miRNA related to invasive growth of nonfunctioning gonadotropic pituitary tumors. Int J Endocrinol (2020) 2020:3730657. doi: 10.1155/2020/3730657 33354213 PMC7737439

[29] Liang S Chen L Huang H Zhi D . The experimental study of miRNA in pituitary adenomas. Turk Neurosurg (2013) 23:721–7. doi: 10.5137/1019-5149.JTN.7425-12.1 24310454

[30] D’Angelo D Palmieri D Mussnich P Roche M Wierinckx A Raverot G . Altered microRNA expression profile in human pituitary GH adenomas: Down-regulation of miRNA targeting HMGA1, HMGA2, and E2F1. J Clin Endocrinol Metab (2012) 97:E1128–38. doi: 10.1210/jc.2011-3482 22564666

[31] Peculis R Niedra H Rovite V . Large Scale molecular studies of pituitary neuroendocrine tumors: Novel markers, mechanisms and translational perspectives. Cancers (Basel) (2021) 13 (6):1395. doi: 10.3390/cancers13061395 PMC8003417 33808624

[32] Moldovan L Batte KE Trgovcich J Wisler J Marsh CB Piper M . Methodological challenges in utilizing miRNAs as circulating biomarkers. J Cell Mol Med (2014) 18:371–90. doi: 10.1111/jcmm.12236 PMC3943687 24533657

[33] Nik Mohamed Kamal NNSB Shahidan WNS . Non-exosomal and exosomal circulatory MicroRNAs: Which are more valid as biomarkers? Front Pharmacol (2019) 10:1500. doi: 10.3389/fphar.2019.01500 32038230 PMC6984169

[34] Endzeliņš E Berger A Melne V Bajo-Santos C Soboļevska K Ābols A . Detection of circulating miRNAs: Comparative analysis of extracellular vesicle-incorporated miRNAs and cell-free miRNAs in whole plasma of prostate cancer patients. BMC Cancer (2017) 17:730. doi: 10.1186/s12885-017-3737-z 29121858 PMC5679326

[35] Kirschner MB Edelman JJB Kao SC-H Vallely MP van Zandwijk N Reid G . The impact of hemolysis on cell-free microRNA biomarkers. Front Genet (2013) 4:94. doi: 10.3389/fgene.2013.00094 23745127 PMC3663194

[36] Mumford SL Towler BP Pashler AL Gilleard O Martin Y Newbury SF . Circulating MicroRNA biomarkers in melanoma: Tools and challenges in personalised medicine. Biomolecules (2018) 8(2):21. doi: 10.3390/biom8020021 PMC6022922 29701682

[37] Kura B Parikh M Slezak J Pierce GN . The influence of diet on MicroRNAs that impact cardiovascular disease. Molecules (2019) 24(8):1509. doi: 10.3390/molecules24081509 PMC6514571 30999630

[38] Tribolet L Kerr E Cowled C Bean AGD Stewart CR Dearnley M . MicroRNA biomarkers for infectious diseases: From basic research to biosensing. Front Microbiol (2020) 11:1197. doi: 10.3389/fmicb.2020.01197 32582115 PMC7286131

[39] Liao C Chen W Fan X Jiang X Qiu L Chen C . MicroRNA-200c inhibits apoptosis in pituitary adenoma cells by targeting the PTEN/Akt signaling pathway. Oncol Res (2013) 21:129–36. doi: 10.3727/096504013X13832473329999 24512727

[40] Wang X Du Q Mao Z Fan X Hu B Wang Z . Combined treatment with artesunate and bromocriptine has synergistic anticancer effects in pituitary adenoma cell lines. Oncotarget (2017) 8:45874–87. doi: 10.18632/oncotarget.17437 PMC5542234 28501857

[41] Vicchio TM Aliquò F Ruggeri RM Ragonese M Giuffrida G Cotta OR . MicroRNAs expression in pituitary tumors: Differences related to functional status, pathological features, and clinical behavior. J Endocrinol Invest (2020) 43:947–58. doi: 10.1007/s40618-019-01178-4 31939196

[42] Cui C Cui Q . The relationship of human tissue microRNAs with those from body fluids. Sci Rep (2020) 10:5644. doi: 10.1038/s41598-020-62534-6 32221351 PMC7101318

[43] Cojocneanu R Braicu C Raduly L Jurj A Zanoaga O Magdo L . Plasma and tissue specific miRNA expression pattern and functional analysis associated to colorectal cancer patients. Cancers (Basel) (2020) 12(4):843. doi: 10.3390/cancers12040843 PMC7226631 32244548

[44] Li S-C Khan M Caplin M Meyer T Öberg K Giandomenico V . Somatostatin analogs treated small intestinal neuroendocrine tumor patients circulating MicroRNAs. PloS One (2015) 10:e0125553. doi: 10.1371/journal.pone.0125553 25942502 PMC4420277

[45] Døssing KBV Kjær C Vikeså J Binderup T Knigge U Culler MD . Somatostatin analogue treatment primarily induce miRNA expression changes and up-regulates growth inhibitory miR-7 and miR-148a in neuroendocrine cells. Genes (Basel) (2018) 9:337. doi: 10.3390/genes9070337 PMC6070923 29973528

[46] Kirschner MB Cheng YY Badrian B Kao SC Creaney J Edelman JJB . Increased circulating miR-625-3p: A potential biomarker for patients with malignant pleural mesothelioma. J Thorac Oncol Off Publ Int Assoc Study Lung Cancer (2012) 7:1184–91. doi: 10.1097/JTO.0b013e3182572e83 22617246

[47] Xu K Chen G Qiu Y Yuan Z Li H Yuan X . miR-503-5p confers drug resistance by targeting PUMA in colorectal carcinoma. Oncotarget (2017) 8:21719–32. doi: 10.18632/oncotarget.15559 PMC5400618 28423513

[48] Yue H-Q Zhou Y-H Guo Y Tang C-Y Wang F Zhou H-D . Serum miR-503 is a candidate biomarker for differentiating metabolic healthy obesity from metabolic unhealthy obesity. Diabetes Metab Syndr Obes (2020) 13:2667–76. doi: 10.2147/DMSO.S262888 PMC7419640 32821139

[49] Li J Xu X Liu C Xi X Wang Y Wu X . miR-181a-2-3p stimulates gastric cancer progression *via* targeting MYLK. Front Bioeng Biotechnol (2021) 9:687915. doi: 10.3389/fbioe.2021.687915 34733825 PMC8558245

[50] Yan W Wang Y Chen Y Guo Y Li Q Wei X . Exosomal miR-130b-3p promotes progression and tubular formation through targeting PTEN in oral squamous cell carcinoma. Front Cell Dev Biol (2021) 9:616306. doi: 10.3389/fcell.2021.616306 33829013 PMC8019696

[51] Dineen R Stewart PM Sherlock M . Acromegaly. QJM (2017) 110:411–20. doi: 10.1093/qjmed/hcw004 26873451

[52] Qin J Li K Wang X Bao Y . A comparative study of functioning and non-functioning pituitary adenomas. Med (Baltimore) (2021) 100:e25306. doi: 10.1097/MD.0000000000025306 PMC8036017 33832102

[53] Lopes MBS . The 2017 world health organization classification of tumors of the pituitary gland: A summary. Acta Neuropathol (2017) 134:521–35. doi: 10.1007/s00401-017-1769-8 28821944

[54] Trouillas J Jaffrain-Rea M-L Vasiljevic A Raverot G Roncaroli F Villa C . How to classify the pituitary neuroendocrine tumors (PitNET)s in 2020. Cancers (Basel) (2020) 12(2):514. doi: 10.3390/cancers12020514 PMC7072139 32098443

