# Supplementary material for: Genome wide analysis of circulating miRNAs in growth hormone secreting pituitary neuroendocrine tumor patients’ plasma
Source: Front Oncol. 2022 Sep 9;12:894317. doi: 10.3389/fonc.2022.894317 (PMC9500360; doi:10.3389/fonc.2022.894317)
Supplement: Supplementary file 1 [file DataSheet_1.zip › Supplementary Figures.docx]

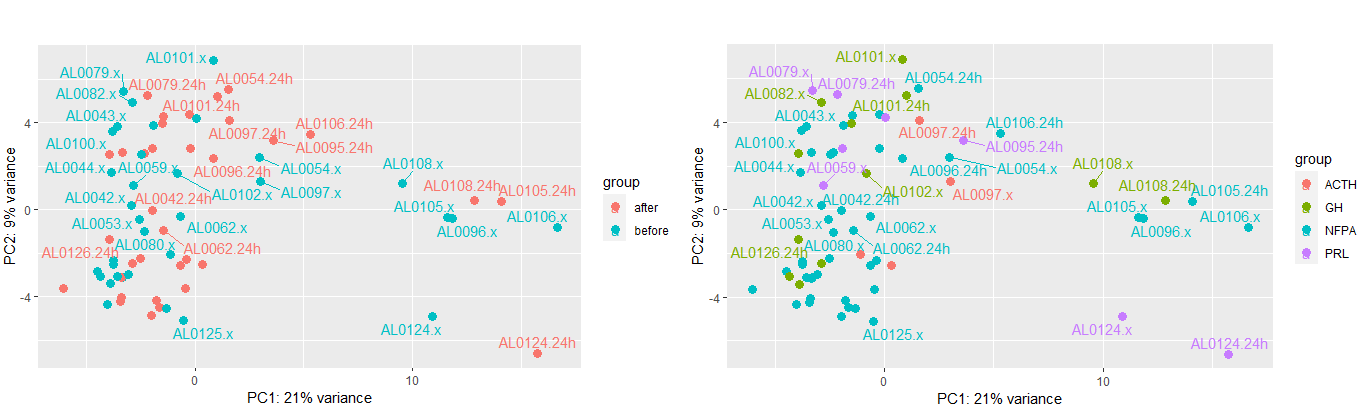


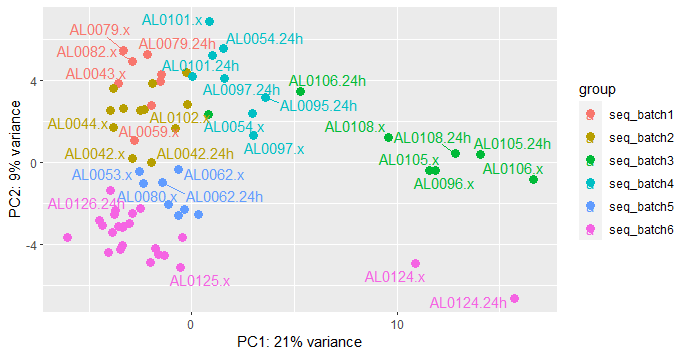


**Supplementary figure 1 –** DESeq2 PCA plots to estimate batch effect of sequened plasma samples.

| P values | P values after “fdrtool” usage |
| --- | --- |
| 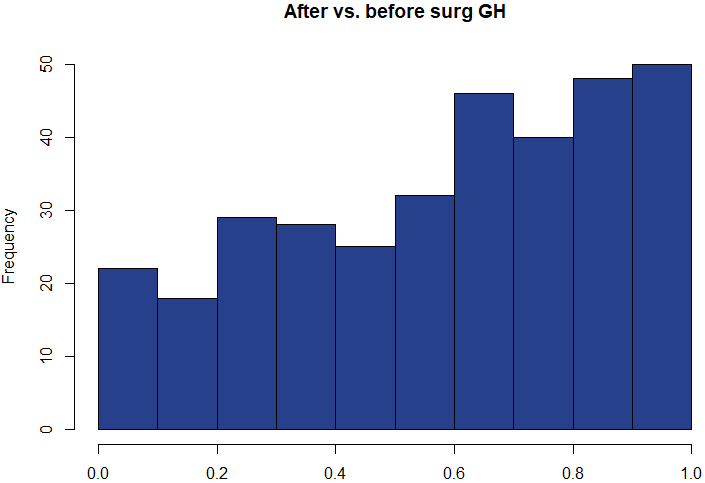 | 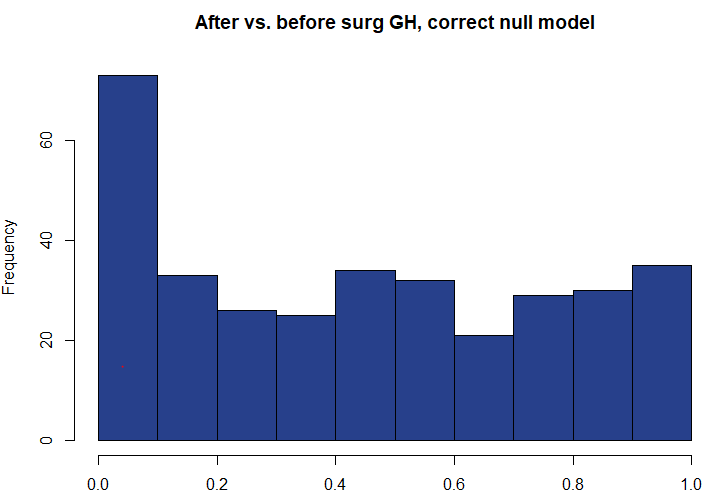 |
| 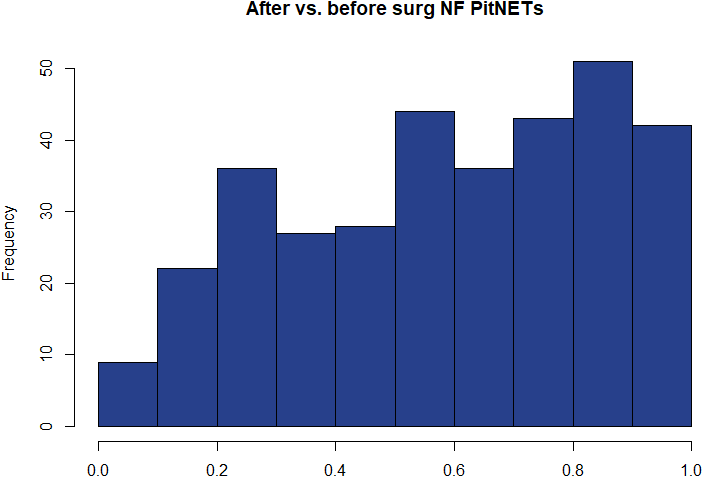 | 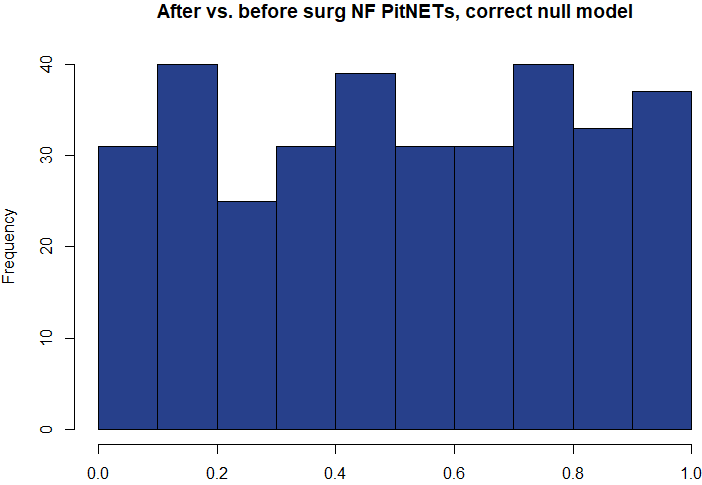 |
| 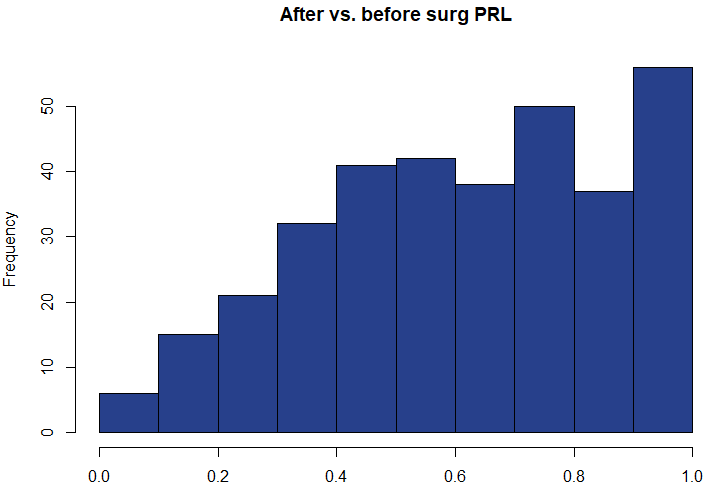 | 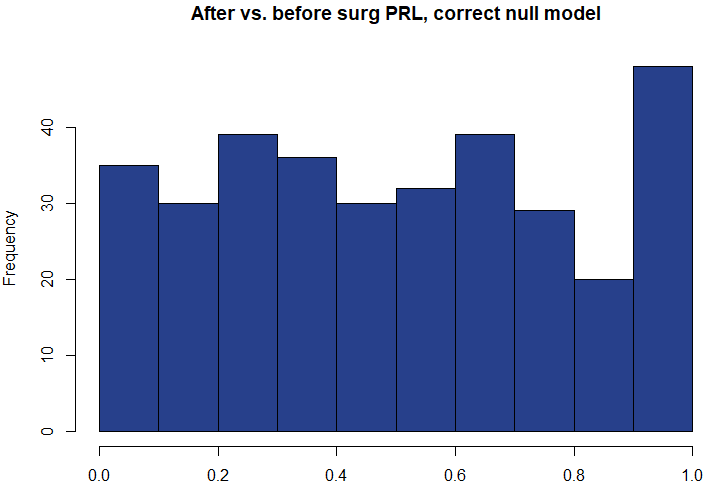 |
| 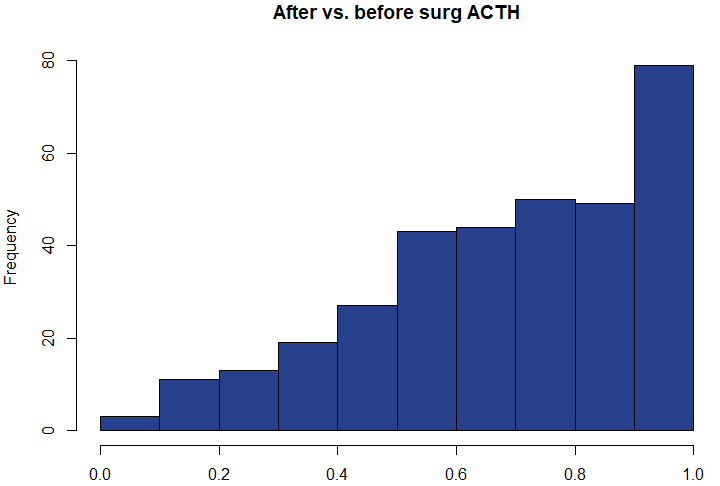 | 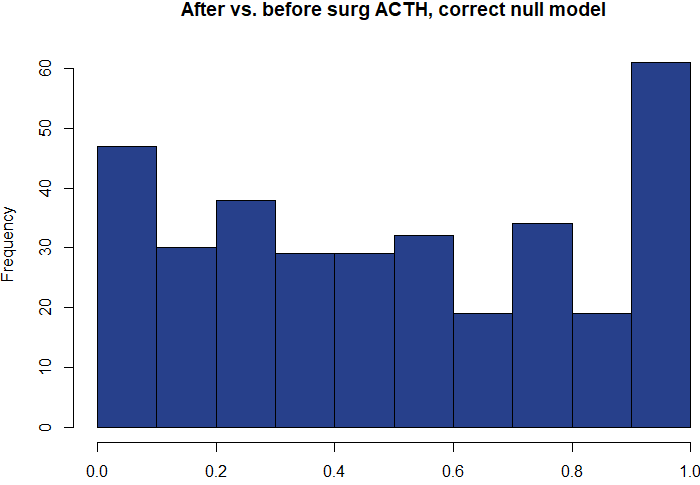 |
| 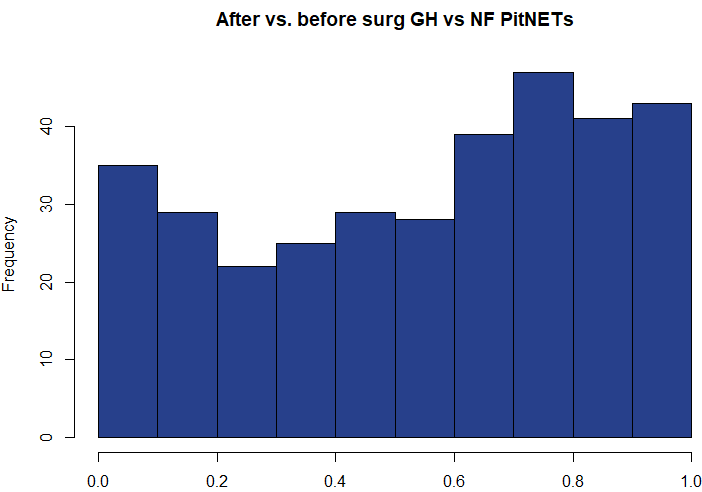 | 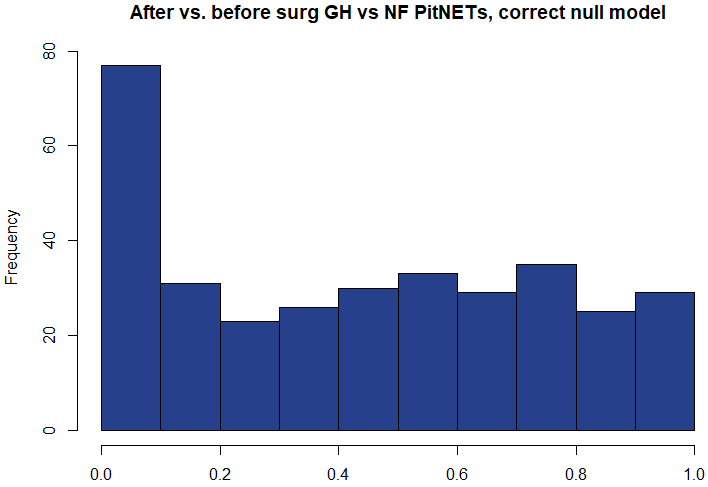 |

**Supplementary figure 2.** DESeq2 P value histograms before and after the usage of “fdrtool”

**Supplementary figure 3**. DESeq2 version 1.30.1 analysis script in R version 4.0.3

| **Before trimming** | **After trimming** |
| --- | --- |
| 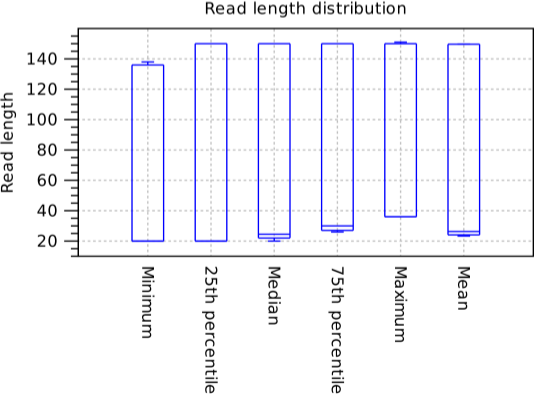 | 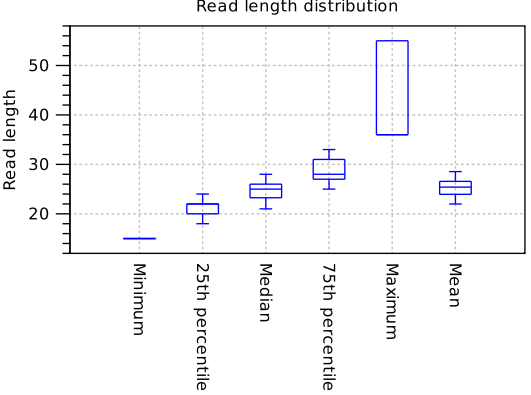 |
| 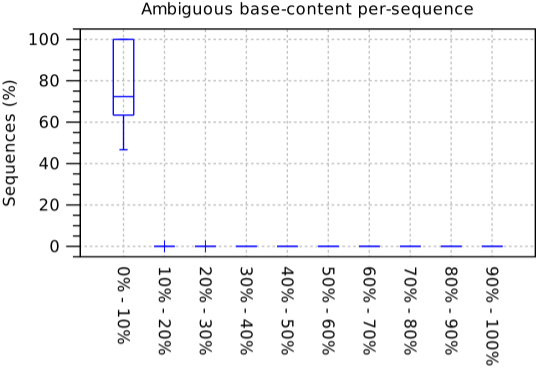 | All reads with ambigous bases were discared.  No data available. |
| 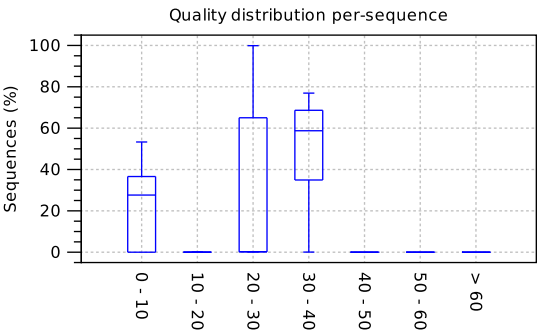 | 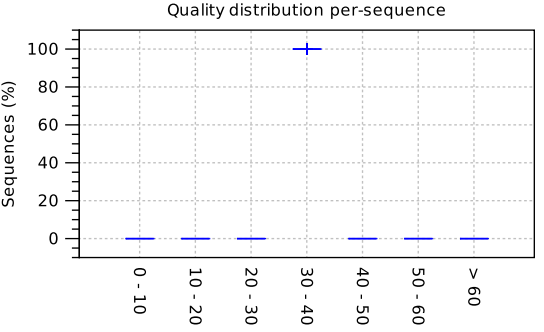 |
| 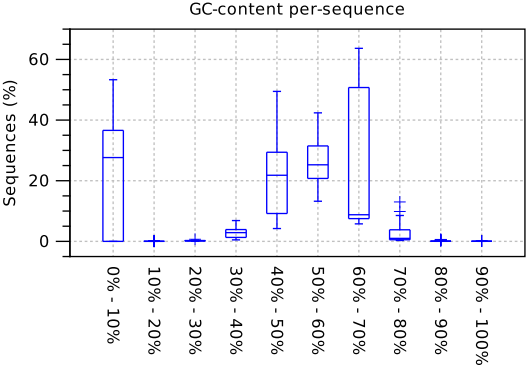 | 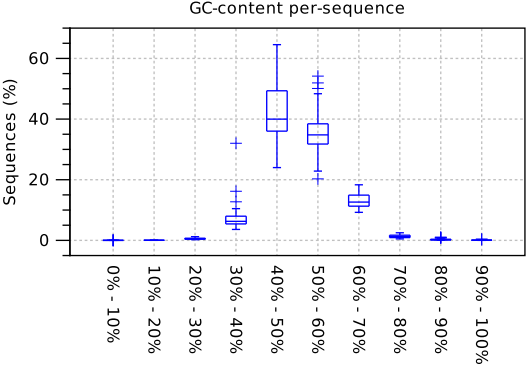 |

**Supplementary Figure 4** – summary of sequence quality statistics before and after trimming for plasma samples.

Read length distribution: The box plots are based on 70 sequenced plasma samples.

Ambiguous base-content: Summarizes the distribution of N-contents. The N-content of a sequence is calculated as the number of ambiguous bases compared to all bases. x: p% - r%: percentage range y: the number of sequences featuring particular N-percentages normalized to the total number of sequences The box plots are based on 70 sequenced plasma samples.

Quality distribution: Summarizes the distribution of average sequence quality scores. The quality of a sequence is calculated as the arithmetic mean of its base qualities. x - y: PHRED range y: the number of sequences observed at that qual. score normalized to the total number of sequences. The box plots are based on 70 sequenced plasma samples.

GC-content: Summarizes the distribution of GC-contents. The GC-content of a sequence is calculated as the number of GC-bases compared to all bases (including ambiguous bases). x: p% - r%: percentage range y: the number of sequences featuring the particular GC-percentage range normalized to the total number of sequences. The box plots are based on 70 sequenced plasma samples.
